# Integrative metabolomics and transcriptomics profiling reveals differential expression of flavonoid synthesis in *Ophiopogon japonicus* (L. f.) Ker-Gawl. in adaptation to drought

**DOI:** 10.1371/journal.pone.0313580

**Published:** 2025-01-07

**Authors:** Tingting Cheng, Juan Lin, Xia Zhou, Hongsu Wang, Xianjian Zhou, Xiaopeng Huang, Tiezhu Chen

**Affiliations:** 1 Sichuan Academy of Chinese Medicine Sciences, Chengdu, China; 2 Hospital of Chengdu University of Traditional Chinese Medicine, Chengdu, China; 3 Sichuan Provincial Key Laboratory of Quality and Innovation Research of Chinese Materia Medica, Chengdu, China; University of California Riverside, UNITED STATES OF AMERICA

## Abstract

Drought is one of the consequences of climate change that severely affects plant growth and development. *Ophiopogon japonicus* (L. f.) Ker-Gawl. (Chinese name: Chuanmaidong, abbreviated as CMD) is a commonly used herbaceous plant whose growth and development are strongly affected by drought. Here, we comprehensively analyzed the transcriptomic and metabolic responses of two CMD varieties (EP and CP) to drought stress. CP utilized a small number of differentially expressed genes to regulate a greater number of differential metabolites compared to EP, suggesting that it may be more drought tolerant. In addition, integrated transcriptome and metabolome analyses revealed that transcription factors such as WRKY, TIFY, and C2H2 regulate flavonoid synthesis in CMD. These findings provide ideas for in-depth analysis of the mechanism of CMD against drought stress, and provide a theoretical basis for breeding high-quality drought-tolerant varieties.

## Introduction

Maidong [*Ophiopogon japonicus* (L.f.) Ker-Gawl], a perennial herbaceous plant in the Liliaceae family [1]. This plant serves as both a medicinal and food source [2–5], primarily composed of flavonoids [6,7], steroidal saponins [8,9], polysaccharides [10,11]and terpenoids [12]. Maidong is primarily found in Sichuan and Zhejiang [13], of which Sichuan Maidong (abbreviated as Chuanmaidong, CMD), which has the advantages of shorter planting cycle, higher yield and cost-effectiveness, is the main variety of Maidong [14]. Currently, although an increasing number of studies have explored the pharmacological and nutritional attributes of CMD, such as alleviation of diseases such as colitis and lung cancer [15,16], and as functional food ingredients [17], there is limited in-depth research on its agronomic properties, in particular on the mechanisms of its responses to adapt to abiotic stresses.

Climate change has exacerbated global water scarcity, making drought stress one of the most detrimental abiotic factors impacting crop production and food security, which is primarily due to agriculture’s heavy reliance on water resources [18,19]. Drought stress disrupts normal plant growth, disturbs metabolic balance, reduces water use efficiency, impairs plant productivity, and poses a significant threat to sustainable crop production [20]. Many agricultural areas are affected by drought, resulting in crop yield losses that can reach up to 50% or even higher [21]. Consequently, uncovering distinctive mechanisms of drought tolerance in plants and screening high quality varieties that can withstand drought stress are urgent issues.

To investigate the mechanisms of CMD adaptation to drought and the ability of different CMD species to adapt to drought, we integrated transcriptomic and metabolomic analyses to identify differentially expressed genes (DEGs), differentially expressed metabolites (DEMs) and differentially expressed transcription factors (DETFs) that adapt CMD to different levels of drought stress, as well as the differences in response to drought stress in different cultivars. Like many other plants, CMD undergoes a complex adaptive process when exposed to drought stress, which may significantly affect the transformation of its secondary metabolites [22,23]. Thus, we also explored the flavonoid regulatory pathways in the drought-adaptation process of CMD. Overall, this study provides a theoretical basis for the selection of optimal drought-resistant CMD varieties and a scientific reference for elucidating the drought tolerance mechanism of CMD.

## Materials and methods

### Plant material and growing conditions

Erect Plants (EP) and Creeping Plants (CP) of CMD varieties were obtained from Sichuan Province, China (31°30’N, 104°95’E and 31°32’N, 104°89’E). These specimens were taxonomically confirmed as *Ophiopogon japonicus* (L. f.) Ker-Gawl. of the Liliaceae family by researcher Dr. Tiezhu Chen. The seedlings were planted in plastic pots and cultivated in the greenhouses at the Sichuan Academy of Chinese Medicine Sciences (30°37’N, 104°40’E). The pots had a caliber of 24 cm, a base diameter of 19.5 cm, and a height of 26.5 cm. The substrate was formulated with an organic matter content of 1.80 g/kg, total N 2.50 g/kg, total P 1.50 g/kg, pH 6.8, and a maximum water holding capacity of the substrate of 25.87%. During the growth and development of the two CMD germplasm resources, the amount of watering was controlled, 16 h/day of light was given and other cultivation and management modes were kept consistent. Seedlings were acclimatized for 28 days, and then watering was controlled. Then, the seedlings of similar height and growth potential were randomly divided into control (CK), moderate drought (D1) and severe drought (D2) groups, with six pots in each group. In the CK group, standard watering practices were maintained, delivering 1598 mL per week, one seventh a day. The standard weekly watering amount is an average of the period of maximum water need (April-June) for CMD, and watering is done once a day. Meanwhile, the D1 group received reduced watering of 799 mL per week, and the D2 group was subjected to even more stringent watering, receiving 559.3 mL per week. After 21 days of drought stress experiment, the plants were dug up and the roots were washed with pure water, then the fresh tubers were quickly cut and placed in liquid nitrogen in preparation for the next experiment. The three samples from each group (CK, D1, D2) of both EP and CP were randomly selected for comprehensive analysis through transcriptomic and metabolomic profiling.

### RNA preparation and RNA-seq

Total RNA was extracted from EP and CP fresh tubers exposed to different drought stress levels, and then RNA libraries were constructed [24,25]. Briefly, mRNA was purified from total RNA using poly-T oligo-attached magnetic beads. Fragmentation was carried out using divalent cations under elevated temperature in NEBNext First Strand Synthesis Reaction Buffer(5X). First strand cDNA was synthesized using random hexamer primer and M-MuLV Reverse Transcriptase. Second strand cDNA synthesis was subsequently performed using DNA Polymerase I and RNase H. Then 3 μL USER Enzyme (NEB, USA) was used with size-selected, adaptor-ligated cDNA at 37°C for 15 min followed by 5 min at 95°C before PCR. Then PCR was performed with Phusion High-Fidelity DNA polymerase, Universal PCR primers and Index (X) Primer [26]. At last, PCR products were purified (AMPure XP system) and library quality was assessed on the Agilent Bioanalyzer 2100 system. Subsequently, sequencing was performed using the Illumina NovaSeq6000 sequencing platform and transcriptome assembly was performed using Trinity [27]. The default setting for min_kmer_cov was applied, while all other parameters were maintained at their default values.

### Transcriptome data analysis

Gene functionality was annotated by referencing several databases, including NR (NCBI non-redundant protein sequences) [28], Pfam (Protein family) [29], KOG/COG/eggNOG (Clusters of Orthologous Groups of proteins) [30], Swiss-Prot (a manually annotated and reviewed protein sequence database) [31], KEGG (Kyoto Encyclopedia of Genes and Genomes) [32], and GO (Gene Ontology) [33]. DEGs among the CK, D1, and D2 groups were identified and analyzed using the DESeq R package (1.10.1), we used the following screening criteria: |fold change| > 2 and FDR < 0.01. The DEGs were subsequently subjected to functional clustering analysis based on the KEGG pathway and GO categories [34].

### Metabolomic profile using liquid chromatography-mass spectrometry

The extraction of total metabolites from fresh tuber of CMD was performed as follows: 50 mg of the sample was weighed, and subsequently, 1000 μL of an extraction solution containing an internal standard (composed of methanol: acetonitrile: water in a 2:2:1, v/v ratio, with an internal standard concentration of 20 mg/L) was added. This mixture underwent vortex mixing for 30 seconds, followed by the addition of steel beads. The sample was then processed using a 45Hz grinder for 10 minutes, with an additional 10-minute ultrasonication step performed in an ice water bath. Afterward, the sample was left to stand at 20°C for 1 hour and subsequently centrifuged at 4°C and 12000 rpm for 15 minutes. Following centrifugation, 500 μL of supernatant was subjected to vacuum drying, followed by the addition of 160 μL of an extraction solution (acetonitrile: water, 1:1, v/v). A 30-second vortex mixing step was performed, followed by 10 minutes of sonication in an ice-water bath. Finally, the sample was centrifuged at 4°C, 12000rpm for 15 minutes.

The metabolites (supernatant, 120 μL) were separated using an LC−MS system (Acquity I-Class PLUS ultra-high performance liquid tandem Waters Xevo G2-XS QTOF high-resolution mass spectrometer). The liquid chromatograph column, Acquity UPLC HSS T3 column 1.8 μm, 2.1 × 100 mm), was purchased from Waters. The metabolite detection ofthe samples was conducted under positive and negative ion modes. The parameters of the ESI ion source were as follows: capillary voltage: 2000 V (positive ion mode) or −1500 V (negative ion mode); cone voltage: 30 V; ion source temperature: 150°C; desolvent gas temperature: 500°C; backflush gas flow rate: 50 L/h; and desolventizing gas flow rate: 800 L/h.

### Metabolomics data analyses

The raw data obtained through MassLynx V4.2 underwent further processing steps, including peak extraction and alignment, utilizing the Progenesis QI software [35]. To assess the reproducibility of the samples, principal component analysis (PCA) was performed. Metabolites were annotated with reference to KEGG, the Human Metabolome Database (HMDB) [36], and lipid profiles. The multiplicity of differences was calculated and compared based on grouping information, and significant differences (p-values) for each compound were determined using the t-test. Additionally, we constructed an OPLSDA model [37] using the R language software package ropls, with model reliability verified through 200 alignment tests. Multiple cross-validation was performed to calculate the VIP value of the model. DEMs were identified using a combination of multiplicity of difference, p-value, and VIP value (FC > 1, p-value < 0.05, VIP > 1). Statistical analysis of metabolome data was performed on the BMKCloud platform (Biotechnology Co., Ltd., Beijing, China).

### Conjoint analysis of transcriptomic and metabolomic data

In the correlation analysis, the relative abundance of genes and the relative abundance of metabolites were used to calculate the correlation coefficients. The key genes and metabolites in the important pathways for drought resistance in CMD were screened separately and characterized using Pearson correlation analysis. The expression of some key enzyme genes in flavonoid metabolism was correlated with the accumulation of flavonoids, and the correlation coefficient, r, indicated the degree of correlation between the two. r closer to 1 indicated a higher degree of positive correlation between the two, while r closer to -1 indicated a higher degree of negative correlation between the two.

### Co-expression network analysis

Co-expression analysis was performed using DETFs detected in EP and CP and DEGs related to flavonoid biosynthesis pathway. The Pearson correlation coefficient (PCC) between DETFs and DEGs was calculated. TF-gene pairs with PCC ≥ 0.90 or ≤ -0.90 were selected for analysis, and the co-expression network was constructed and visualized using Cytoscape software.

### Real-time quantitative PCR validation

Seven genes were randomly selected for qRT—PCR analysis. Specific primers were designed using Primer Premier 5.0 software, details are shown in S1 File. qRT—PCR was carried out in 20 μL reaction, 2×ChamQ SYBR Color qPCR Master Mix 10 μL, 2 μL each of upstream and downstream primers, 4 μL of template cDNA, and ddH_2_O was added to 20 μL. The reaction parameters were as follows: 30 s at 95°C, 40 cycles of 15s at 95°C, and 30s at 60°C. The relative expression of mRNA was calculated using 2^-ΔΔCt^, and all experiments were repeated three times [38].

### Statistical analysis

Excel 2019 software was used for statistical analysis. qRT-PCR validated bar graphs show the mean ± SD of three independent experiments.

## Results

### Analysis of the differentially expressed genes (DEGs)

**Fig 1:**
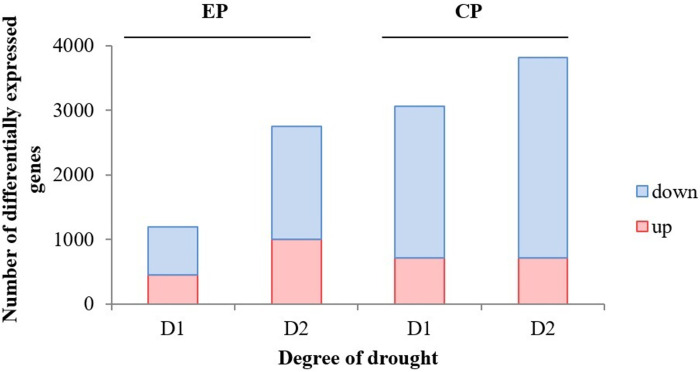
Number of differentially expressed genes after D1 and D2 drought treatments. Red bars indicate an increase, blue bars indicate a decrease.

This study yielded a total of 112.15 GB of clean data from the 18 samples, with each sample demonstrating a Q30 base percentage exceeding 92.83%, indicating high data quality. Following the assembly process, we obtained a set of 37,247 Unigenes. Of these, 32,525 Unigenes received annotations, and notably, 22,167 Unigenes exceeded the 1 kb length threshold, further affirming the robustness of our transcriptome dataset. The resulting data are visually presented in Fig 1. Notably, the total count of DEGs showed an increasing trend in both EP and CP with higher levels of drought stress post-treatment. However, the extent of this increase varied between the two varieties. Specifically, DEGs in EP showed a substantial 130% increase, with the number of up-regulated genes rising from 449 to 1003 and down-regulated genes from 742 to 1747. In contrast, CP displayed a relatively stable count of up-regulated genes, while the number of down-regulated genes increased from 2338 to 3110. These findings highlight that drought stress treatment induced notable transcriptome alterations in CMD tissues, with the total number of DEGs positively correlated with the severity of drought.

### Differential gene function analysis

**Fig 2:**
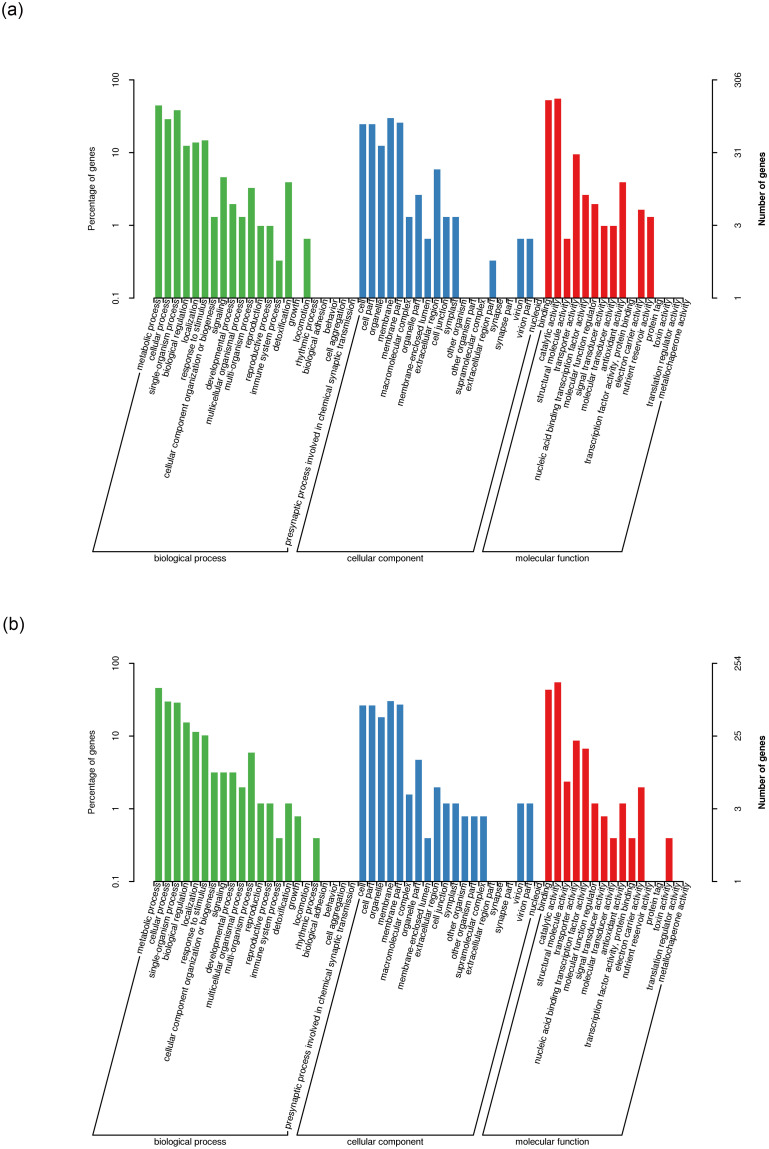
Categorization of differentially expressed genes in GO for the D1 and D2 treatment groups. (a) GO enrichment in the D1 treatment group, (b) GO enrichment in the D2 treatment group.

To assess the biological functions of the differential genes under drought stress treatments, we separately annotated the D1 and D2 treatment groups’ differential genes using the GO database, categorizing them into biological processes, cellular components and molecular functions. Notably, as shown in Fig 2, the top three major enriched categories were consistent in both D1 and D2, encompassing metabolic processes, cellular processes and single-organism processes in the biological process category, membrane-related components in the cellular component category, and catalytic activities, binding and transporter activities in the molecular function category. However, differences were also observed between D1 and D2 as they exhibited distinct enriched categories. D1 was characterized by enrichments related to locomotion, extracellular region parts and nutrient reservoir activities, while D2 did not exhibit these specific enrichments. Conversely, D2 displayed enrichments associated with growth, other organism parts, supramolecular complexes, transcription factor activities, protein binding and toxin activities, which were not observed in D1.

**Fig 3:**
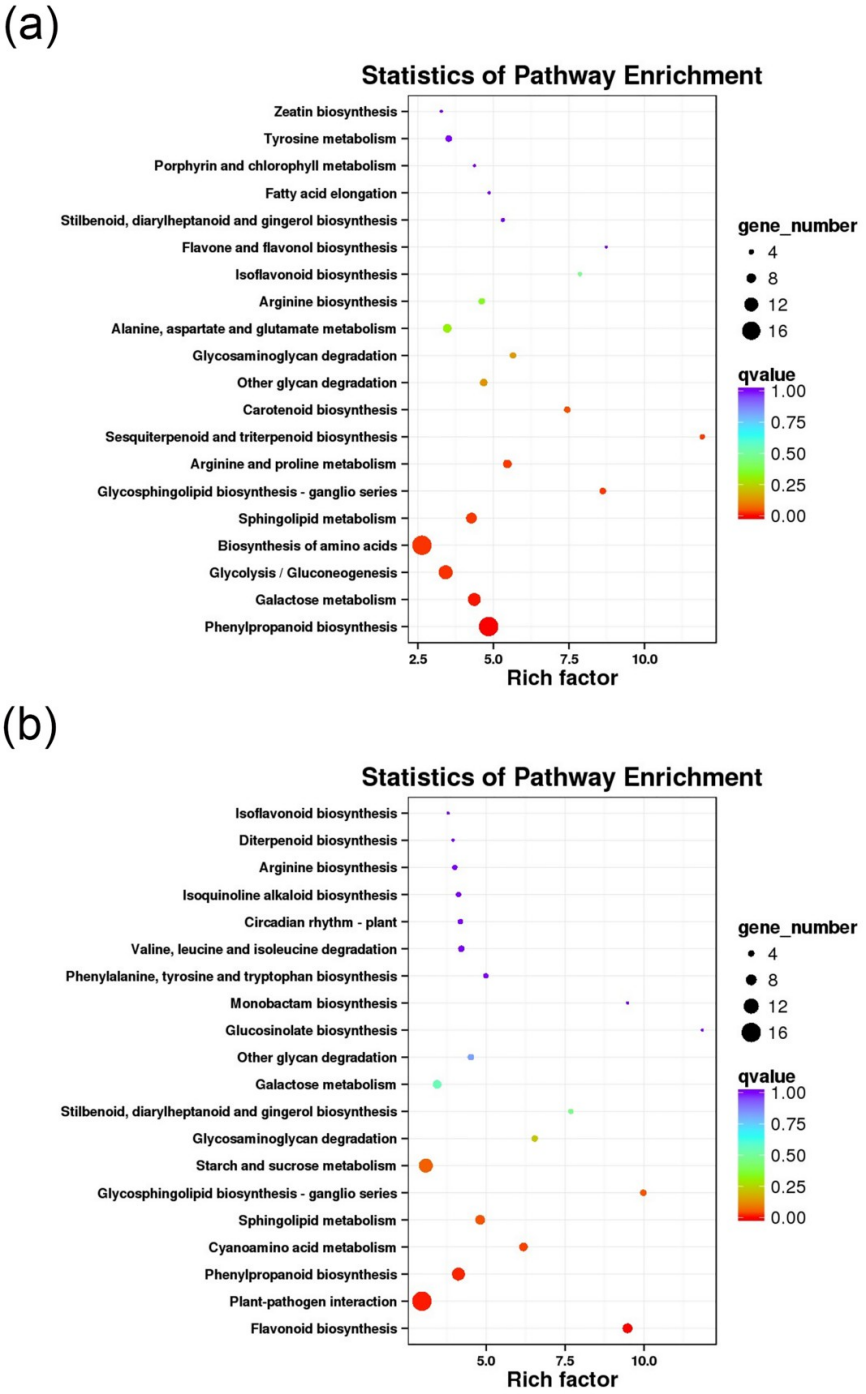
KEGG enrichment maps of differentially expressed genes in the D1 and D2 treatment groups. (a) KEGG enrichment in the D1 treatment group, (b) KEGG enrichment in the D2 treatment group.

The KEGG enrichment analysis results show distinct pathway enrichments in response to drought stress treatments (Fig 3). Specifically, in the D1 group, we found enrichment of differential genes in pathways related to the biosynthesis of amino acids, phenylpropanoid biosynthesis and glycolysis/gluconeogenesis (carbon metabolism). On the other hand, in the D2 group, differential genes exhibited enrichment in pathways associated with plant-pathogen interaction, starch and sucrose metabolism, and phenylpropanoid biosynthesis. Significantly, both the D1 and D2 groups displayed substantial enrichment in the phenylpropanoid biosynthesis pathway, which serves as a crucial source for flavonoid biosynthesis—the primary active ingredient in CMD. Furthermore, the D2 group exhibited a significant enrichment in the flavonoid biosynthesis pathway. These findings strongly suggest that flavonoids represent a pivotal pathway in the adaptation of CMD to drought stress.

### Metabolomic analysis

**Fig 4:**
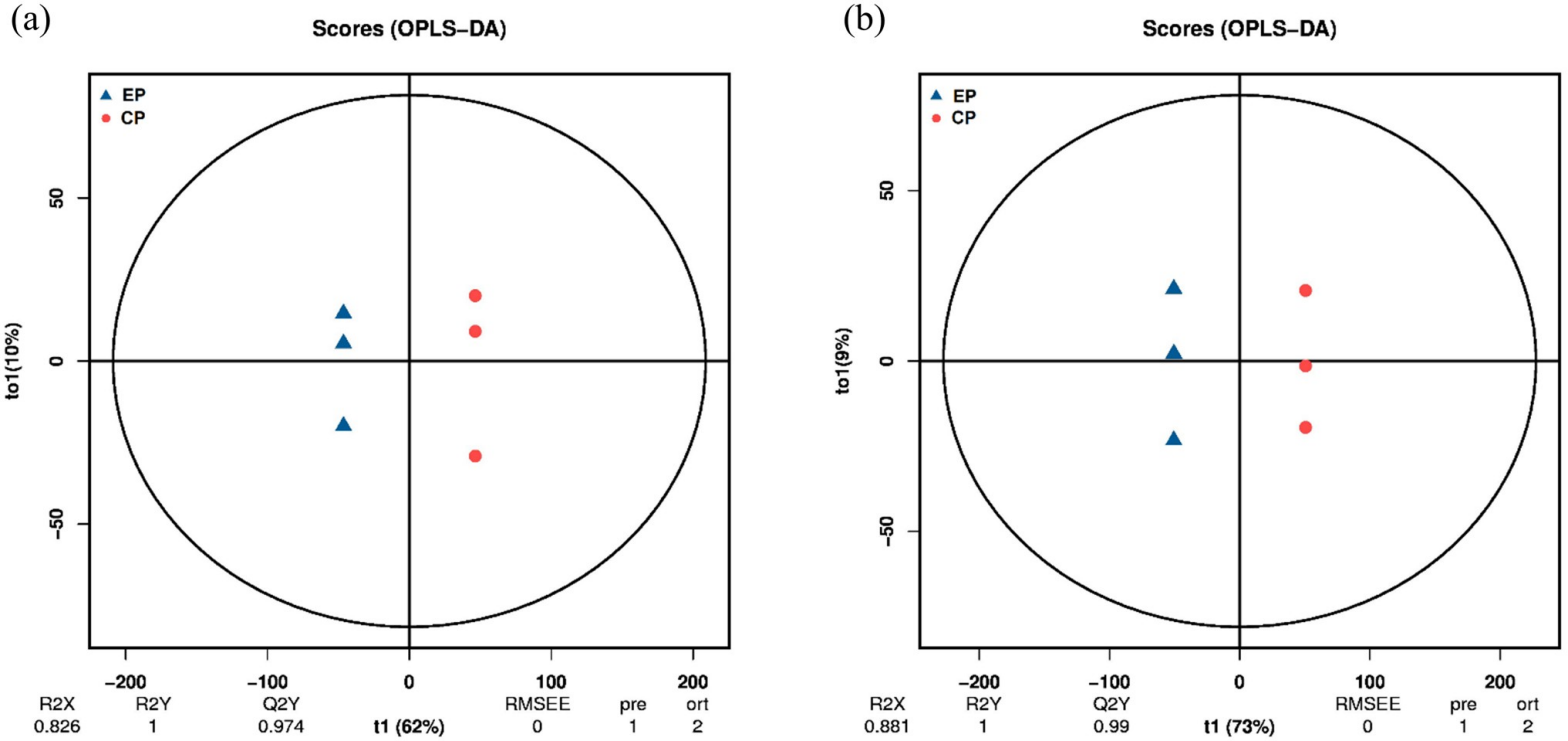
OPLS-DA analysis of metabolites identified in D1 and D2. Blue triangles indicate EPs, red circles indicate CPs. (a) OPLS-DA analy-sis in the D1 treatment group, (b) OPLS-DA analysis in the D2 treatment group.

To investigate the inherent material distinctions between EP and CP in their responses to drought, we used metabolomics for the identification of differential metabolites. Three samples were taken from each treatment group for metabolomic analysis. PCA demonstrated a degree of data consistency among replicate samples within each group (S2 File), effectively showcasing the clear segregation of metabolites between the different CMD types. Furthermore, the OPLS-DA results yielded robust Q2Y values of 0.97 and 0.99, respectively (Fig 4). These high Q2Y values indicate the reliability and consistency of the metabolomics data. Metabolite identification in this study was based on metabolite data detected in both positive and negative ion modes, with a total of 23,642 peaks detected, of which 4,240 metabolites were annotated.

#### The abundance of metabolites differed between EP and CP acclimatized to drought

**Fig 5:**
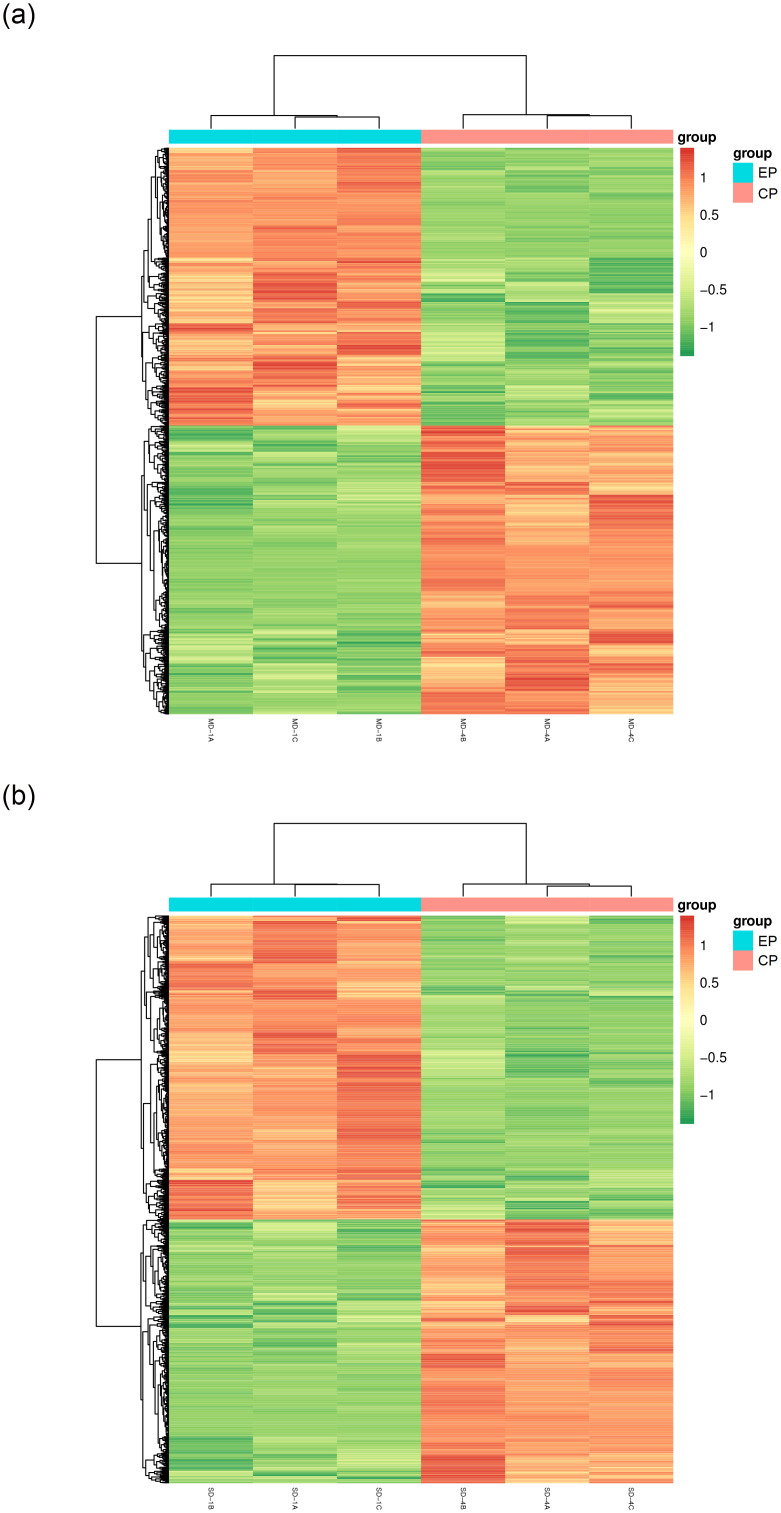
Heatmap of clustering of differentially expressed metabolites for D1 and D2. (a)D1 treatment group, (b)D2 treatment group.

To investigate the differences in drought adaptation between EP and CP, we quantitatively analyzed and compared the measured metabolites. In the D1 treatment group, we identified 2,124 DEMs, with 1,103 up-regulated and 1,021 down-regulated metabolites. In the D2 treatment group, we found 2,691 DEMs, consisting of 1,274 up-regulated and 1,417 down-regulated metabolites (S3 File). Notably, as drought severity increased, both EP and CP exhibited an increase in the total number of DEMs, up-regulated DEMs, and down-regulated DEMs. Specifically, the total DEMs increased by 366 and 489, up-regulated DEMs by 238 and 253, and down-regulated DEMs by 128 and 236, respectively, in EP and CP. The clustered heat map of differential metabolites, as shown in Fig 5, indicates a high level of reproducibility among samples within each group, reinforcing the robustness of our findings.

#### EP and CP have different metabolic patterns of flavonoids during drought acclimation

**Fig 6:**
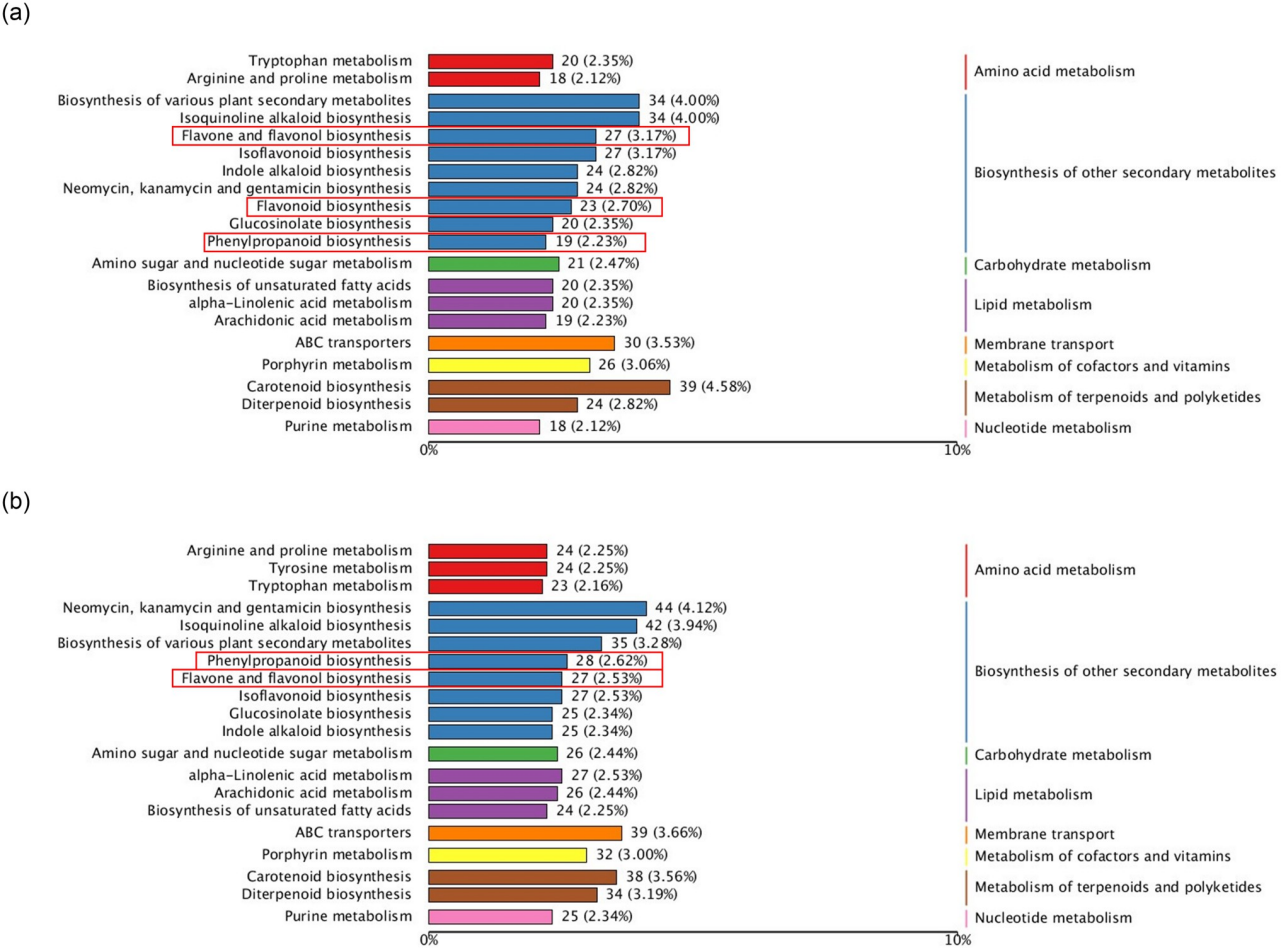
Top 20 enriched metabolite categories based on the HMDB database. (a)D1 treatment group, (b)D2 treatment group. Sections boxed in red indicate flavonoid-related metabolic pathways.

**Fig 7:**
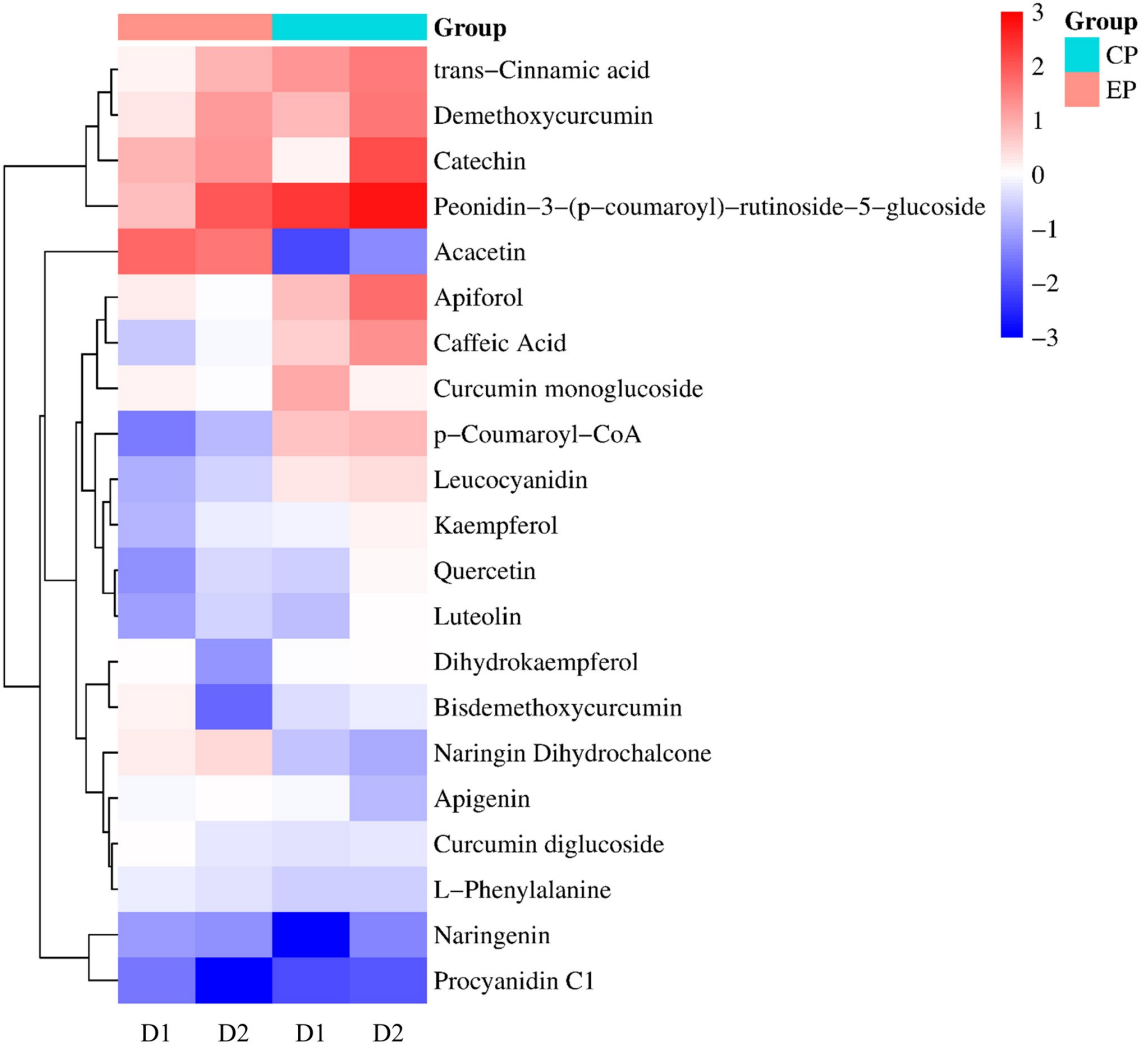
Expression profiles of differentially expressed genes for flavonoid biosynthesis. The expression profile of each gene was determined based on log2(FC) in the RNA-seq data.

Many bioactive compounds are synthesized through phenylpropanoid metabolism, among which flavonoids are a crucial branch of this pathway. During drought adaptation, different CMD varieties accumulated substantial quantities of flavonoid secondary metabolites (Fig 6). Our metabolomic analysis identified several DEMs within the flavonoid biosynthesis pathway, including demethoxycurcumin, catechin, peonidin derivatives and Apiforol, among others (Fig 7). Notably, the accumulation patterns of flavonoids in EP and CP differed significantly. For instance, in EP, acacetin levels were found to be increased, while naringin derivatives (specifically naringin dihydrochalcone) were increased in EP but decreased in CP. Similarly, Leucocyanidin and kaempferol increased in CP but decreased in EP. These distinct patterns highlight the contrasting responses of EP and CP to drought stress, particularly concerning the accumulation of flavonoid metabolites.

### Expression of flavonoid synthesis-related genes in two CMD varieties

**Fig 8:**
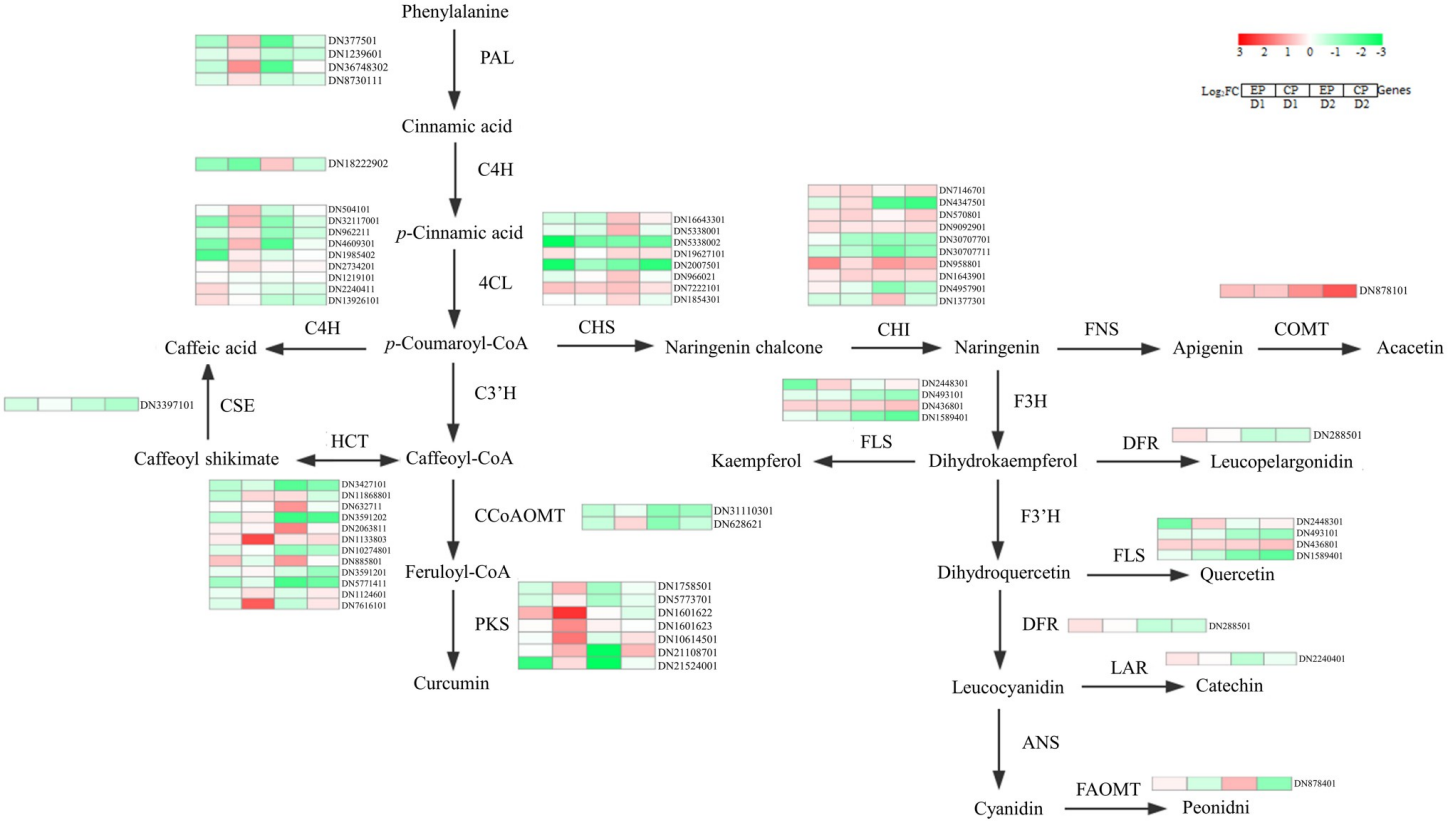
Differences in flavonoid metabolism between EP and CP under different extents of drought. The level of transcription of the enzyme is represented by a heat map. The genes encoding enzymes include phenylalanine ammonia-lyase (PAL), cinnamate 4-hydroxylase (C4H), 4-coumarate-CoA ligase (4CL), p-coumaroyl quinate/shikimate 3’-hydroxylase (C3’H), caffeoyl-CoA-O-methyltransferase (CCoAOMT), polyketide synthase (PKS), caffeoyl shikimate esterase (CSE), shikimate O-hydroxycinnamoyltransferase (HCT), chalcone synthase (CHS), chalcone isomerase (CHI), flavone synthase (FNS), caffeic acid-O-methyltransferase (COMT), flavone 3-hydroxylase (F3H), flavonol synthase (FLS), dihydroflavonol reductase (DFR), flavonoid 3’-hydroxylase (F3’H), leucoananthcyanidin reductase (LAR), anthocyanidin synthase (ANS), flavonoid 3’, 5’methyltransferase (FAOMT).

To investigate the flavonoid accumulation mechanisms during CMD’s adaptation to drought, we constructed a gene expression heat map focusing on enzymes in the flavonoid biosynthesis pathway based on literature findings and RNA-seq data. Fig 8 illustrates our key observations, whereby in CP, D1 treatment led to an overall upregulation of PAL, 4CL, HCT and PKS gene expression. Consequently, this upregulation indirectly elevated the levels of trans-cinnamic acid, caffeic acid and curcumin derivatives (specifically, demethoxycurcumin and curcumin monoglucoside) (Fig 7). Additionally, p-coumaroyl-CoA levels, a direct precursor in flavonoid biosynthesis, were increased. Conversely, in EP, the D2 treatment resulted in overall increased CHS gene expression, indirectly leading to elevated levels of naringenin chalcone derivatives, specifically naringin dihydrochalcone. These findings shed light on how changes in gene expression contribute to the modulation of flavonoid accumulation in CMD during drought adaptation.

### Correlation analysis between key DEGs expression and accumulation of flavonoids

**Fig 9:**
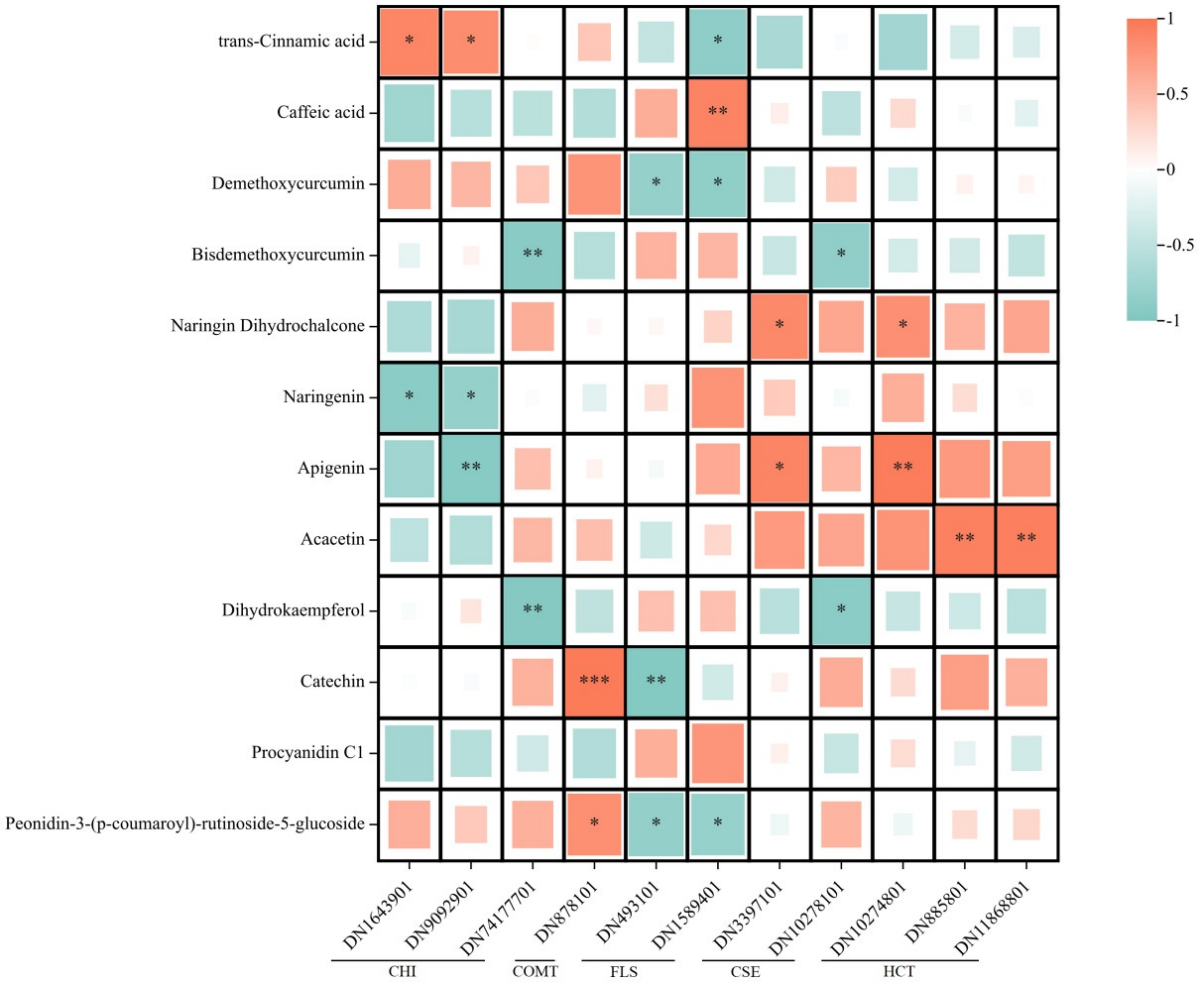
Pearson correlation analysis between the differentially expressed genes and differentially expressed metabolites in the flavonoid biosynthesis pathway. Significant correlations are marked with * (p < 0.05) and ** (p < 0.01). Red color indicates positive correlation and green color indicates negative correlation.

Pearson correlation analysis was conducted to examine the relationship between the expression levels of DEGs and the contents of compounds within the flavonoid biosynthetic pathway (Fig 9). The results revealed significant correlations between the compounds in the synthesis pathway and the associated DEGs. Specifically, CHI (DN9092901) displayed a negative correlation with naringenin and apigenin (r < -0.83) while exhibiting a positive correlation with trans-cinnamic acid (r > 0.84). Consequently, the expression of CHI in CP was higher than that in EP, but the content of naringenin and apigenin was lower in CP than in EP. The expression of CSE (DN3397101) and HCT (DN10274801) was positively correlated (r < -0.83) with naringin dihydrochalcone, and the content of apigenin exhibited a positive correlation (r > 0.81) with these genes. Additionally, the accumulation of acacetin was positively correlated (r > 0.93) with HCT (DN885801 and DN11868801). Furthermore, COMT (DN878101) showed a positive correlation (r > 0.82) with the content of catechin and peonidin derivatives, while FLS (DN493101) displayed a negative correlation with these compounds (r < -0.84). These correlation findings provide valuable insights into the interplay between gene expression and the accumulation of specific flavonoid compounds in CMD during drought adaptation.

### Gene regulatory networks of flavonoids

**Fig 10:**
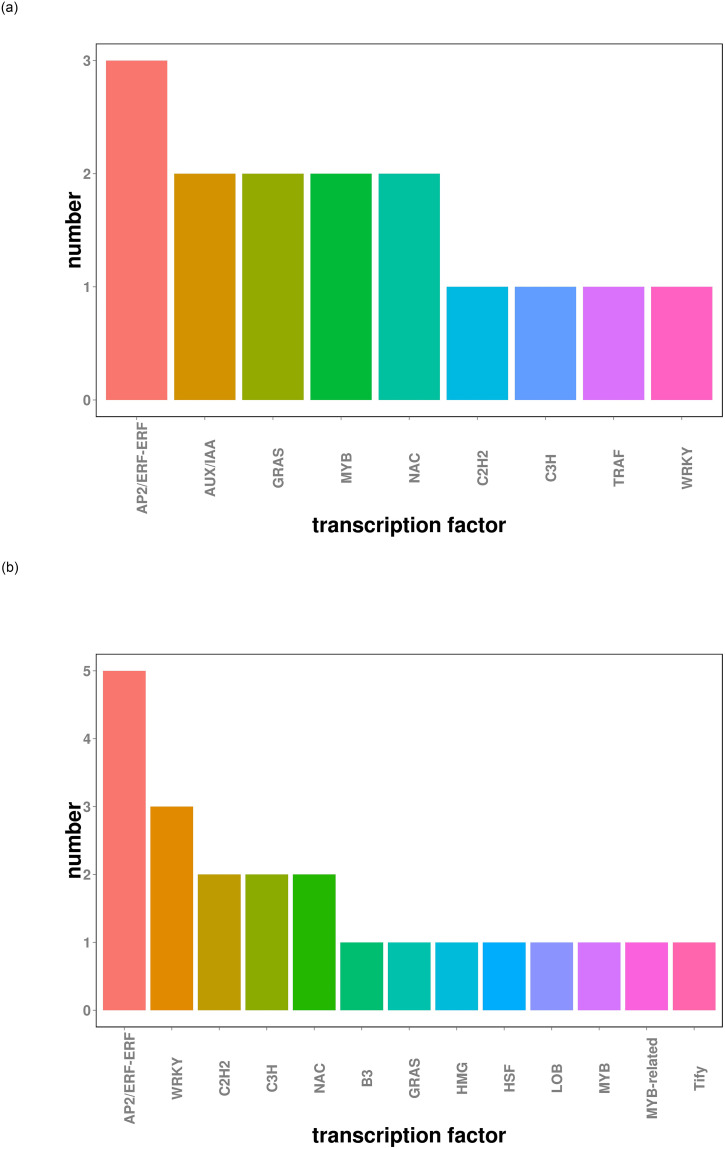
Differentially expressed transcription factors in D1 and D2. (a) D1 treatment group, (b) D2 treatment group.

**Fig 11:**
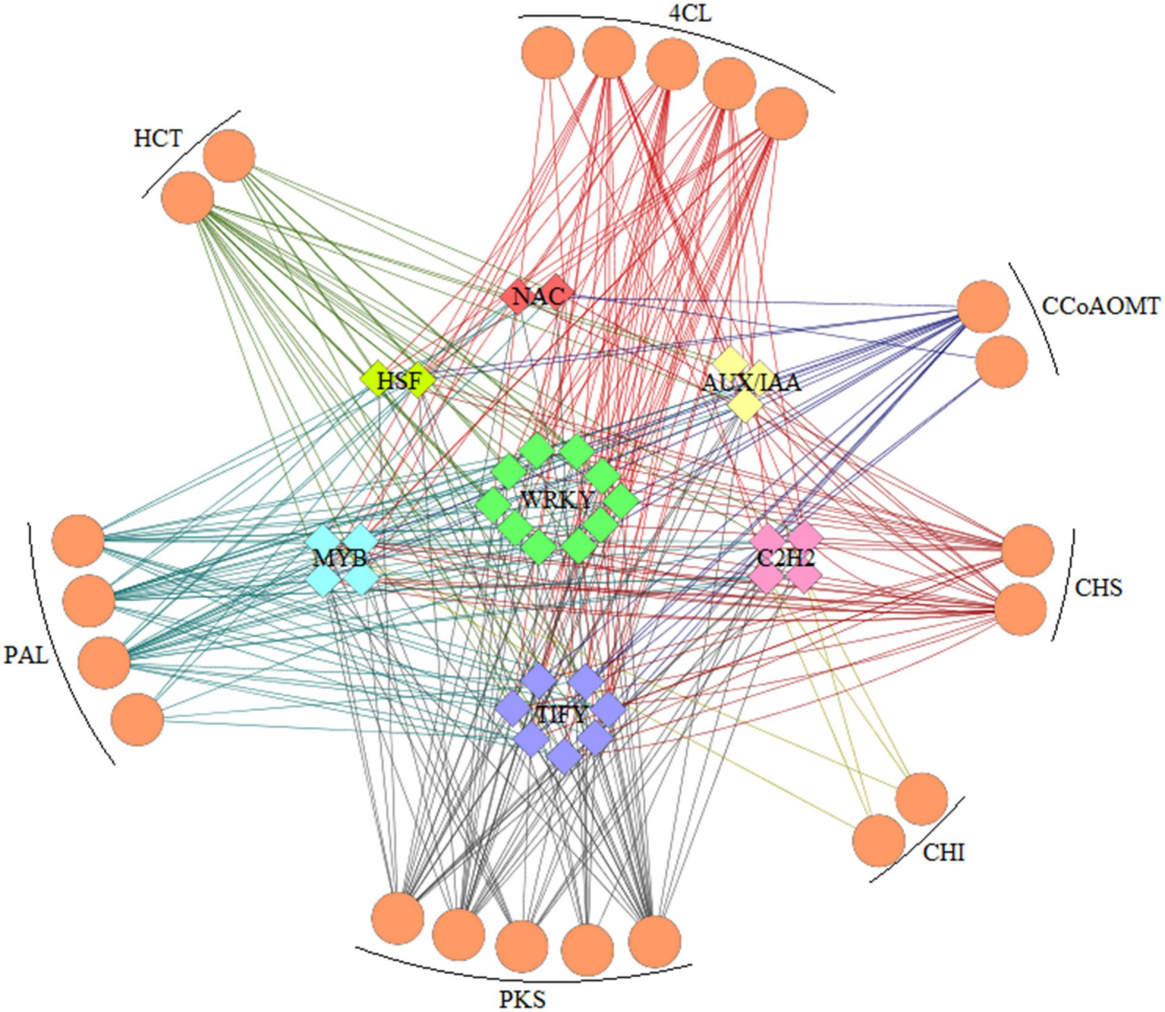
Potential interactions of differentially expressed transcription factors with key genes in the flavonoid biosynthetic pathway. Co-expression networks connecting structural genes in flavonoid biosynthesis (circles) with TFs (diamonds).

Structural genes involved in flavonoid metabolism are typically under the regulatory control of transcription factors (TFs). Herein, a variety of TFs were identified among the DEGs in drought-stressed CMD (Fig 10), including AP2/ERF-ERF, AUX/IAA, GRAS, MYB, NAC, C2H2, C3H, TRAF, WRKY, B3, HMG, HSF, LOB and Tify. The number and types of TFs varied considerably across different drought levels. In the D1 treatment, the top 5 TFs were AP2/ERF-ERF, AUX/IAA, GRAS, MYB and NAC, while under D2 treatment, the top 5 TFs were AP2/ERF-ERF, WRKY, C2H2, C3H and NAC. To explore the relationships between these TFs and key enzyme genes, Pearson correlation analysis was conducted based on the LOG2FC of the transcripts of the DETFs and the key enzyme genes. We selected correlations with Pearson correlation coefficients |r| ≥ 0.9 and p < 0.05 for visualization purposes (Fig 11). The results demonstrated that DEGs encoding 4CL, PAL, PKS, CHS, HCT, CCoAOMT and CHI exhibited co-expression with various DETFs. The expression of these genes was regulated by numerous TFs, including WRKY, TIFY, MYB, C2H2, AUX/IAA, NAC and HSF. Overall, these findings elucidate the complex regulatory networks involving TFs and key enzyme genes in CMD’s response to drought stress, shedding light on the intricacies of flavonoid metabolism regulation.

### Validation by qRT-PCR

**Fig 12:**
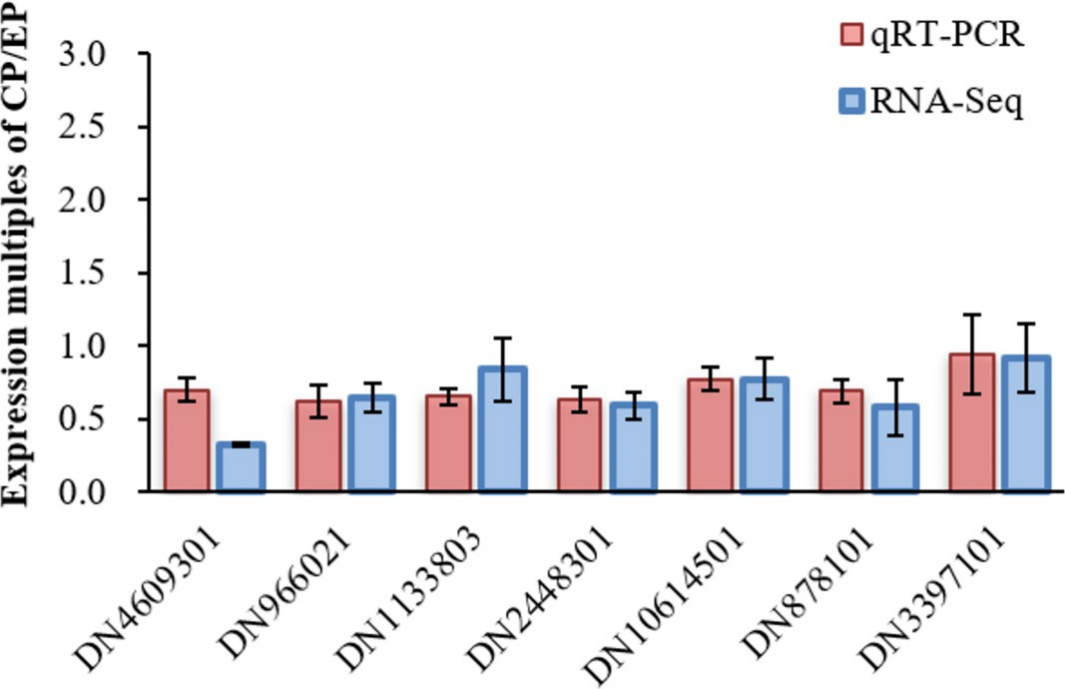
qRT-PCR verification of some key genes with RNA-seq expression. Red bars indicate multiplicity of cp/ep expression in RNA-Seq; blue bars indicate multiplicity of cp/ep expression in qRT-PCR. Data are mean ± standard deviation (SD) (three biological replicates and three technical repeats).

To verify the accuracy of DEGs, seven differentially expressed genes (DN4609301(4CL), DN966021(CHS), DN1133803(HCT), DN2448301(F3H), DN10614501(PKS), DN878101(FAOMT), DN3397101(CSE)) were randomly selected from flavonoid metabolism (Fig 8) for qRT—PCR validation. As shown in Fig 12, there was no significant difference between the fold expression of CP and EP in qRT—PCR and RNA -seq, which highly confirmed the credibility of the RNA -seq results.

## Discussion

CMD is rich in nutrients and medicinal components, and is the raw material of many health products and proprietary Chinese medicines, which are in great demand in the market. Currently, the main focus is on the pharmacological effects of CMD, and few people have studied its abiotic resistance. Here, we comprehensively analyzed the transcriptomics and metabolomics of CMD under drought stress to reveal the differences in the adaptability of different CMD varieties to drought, particularly in terms of flavonoid biosynthesis and oxidase regulation. In the following, we discuss the functional categorization of gene expression and metabolites with varying abundance, which could serve as a valuable resource for enhancing our understanding on the intricate metabolic variations occurring during drought acclimation in different CMD varieties.

Metabolic regulation plays a pivotal role in enabling plants to uphold cellular osmotic potential during drought stress, with plant metabolic genes and metabolites participating in numerous metabolic pathways to adapt to such stressors [39]. For instance, previous research has revealed significant alterations in metabolic pathways, including the TCA cycle and glycolytic pathway, in response to drought stress in different soybean varieties, with notable transcriptional changes occurring in numerous genes [40]. Sprenger et al. similarly observed strong drought-induced responses in transcripts across different potato varieties, with more pronounced transcriptional changes detected in the sensitive variety [41]. In CMD, as drought progressed from moderate to severe, DEGs increased by 130% and DEMs by 366 in EP. In contrast, CP exhibited a 25% increase in DEGs and a 489 increase in DEMs under the same drought conditions. From this, it can be seen that drought stress increased the number of DEGs and DEMs, and the extent of the increase was strongly correlated with the severity of drought and the type of CMD, which is similar to the results of the above references. Our findings suggest that CP efficiently mobilizes a smaller number of genes to regulate a larger array of metabolites, indicating a more effective response to drought compared to EP and underscoring CP’s superior drought tolerance compared to EP.

Flavonoids, a category of polyphenolic compounds renowned for their antioxidant properties, have been implicated in the regulation of plant tolerance to diverse abiotic stressors. Recent investigations, such as the work conducted by Zhao et al. through transcriptome and metabolome analyses, suggest that phenylpropanoid/flavonoid metabolism in soybeans could serve as a pivotal metabolic pathway for drought tolerance in crops [42]. Similarly, Gai et al. demonstrated that elevating the levels of flavonoids, anthocyanins, flavonols and isoflavonoids could enhance drought tolerance in tea trees [43]. In regard to white clover, the accumulation of quercetin and kaempferol in response to water deficit has been linked to increased drought tolerance [44]. Furthermore, studies on rice have identified 4-hydroxycinnamic acid and ferulic acid as critical metabolites for drought tolerance, with DEGs involved in the biosynthesis of these metabolites emerging as promising candidate genes for enhancing drought tolerance [45]. We observed a large enrichment of DEGs in the phenylpropanoid biosynthetic pathway and flavonoid biosynthetic pathway under drought conditions. In particular, most metabolites in the flavonoid biosynthesis pathway in CP showed an upward trend and increased with increasing drought, including trans-cinnamic acid, caffeic acid and curcumin derivatives. This suggests that these substances may be key metabolites for CP adaptation to drought environments, and DEGs involved in the synthesis of these compounds may be able to contribute to the improvement of CMD drought tolerance. In addition, these metabolites have various biological activities [46–48], we can try to subject CMD to moderate drought stress to increase the content of its active ingredients to improve its medicinal and commercial value.

Transcription factors play pivotal roles in plants’ responses to drought stress. Existing literature highlights several examples of their involvement in enhancing drought tolerance. For instance, SbWRKY30 has been shown to regulate the drought stress response gene SbRD19 in sorghum, thus enhancing drought tolerance by increasing the capacity to scavenge reactive oxygen species (ROS) and protect plant cells [49]. In walnuts, the expression of TIFYs has been linked to the regulation of drought tolerance [50]. C2H2-type zinc finger proteins have demonstrated the ability to enhance drought tolerance in transgenic plants by elevating the levels of osmoregulatory substances [51]. MYB proteins, a crucial family of transcription factors, have been associated with improved drought tolerance by regulating flavonoid synthesis and cuticle formation, impacting leaf permeability [52]. Overexpression of Aux/IAA proteins has been found to modulate the ABA signaling pathway, enhance resistance to oxidative stress, and improve drought tolerance in Brachypodium [53]. Additionally, the NAC transcription factor has been shown to increase leaves’ water content, reduce hydrogen peroxide levels, and act as a regulator of drought tolerance in tomatoes [54]. HSF transcription factors promote flavonoid accumulation, ROS scavenging, abscisic acid-induced stomatal closure, and the expression of abscisic acid signaling-related genes under drought conditions, contributing to enhanced plant survival [55]. The co-expression network we constructed provides insights into the crucial roles of TFs in regulating gene expression in CMD. Numerous types of TFs have been reported to be intricately involved in the regulation of key genes responsible for flavonoid synthesis, including a range of families, such as WRKY, TIFY, C2H2, MYB, AUX/IAA, NAC, HSF, among others. Specially, the transcription factors WRKY and TIFY are prominent in the co-expression network (Fig 11). Metabolome and transcriptome co-expression analysis of two different disease resistance genotypes of *P*. *edulis* identified PeWRKY30 as a key TF co-expressed with flavonoid accumulation in yellow fruit *P*. *edulis* [56]. TIFY transcription factors in Tartary Buckwheat and walnut respond to drought stress and a variety of other abiotic stresses [50–57]. Those suggests that WRKY and TIFY transcription factors may also play a central role in CMD adaptation to drought stress. Notably, a single TF can control the synthesis of multiple proteins associated with flavonoids in CMD, and conversely, each protein can be influenced by several TFs. These intricate interactions illustrate the complexity of the regulatory mechanisms associated with flavonoid synthesis in CMD, urging the need for further experimental investigations to fully elucidate these relationships.

## Conclusion

This study represents comprehensive exploration of transcriptomic and metabolomic responses in CMD under varying drought conditions. Drought stress induced notable changes in both gene expression and metabolite levels in CMD. We observed a notable enrichment of DEGs in the phenylpropanoid biosynthesis pathway, accompanied by an accumulation of flavonoid-related metabolites, particularly in creeping CMD. Furthermore, our investigation highlights the pivotal role of transcription factors, including WRKY, TIFY, C2H2, MYB, AUX/IAA, NAC and HSF, in regulating flavonoid synthesis in CMD. Interestingly, creeping CMDs probably displayed superior drought tolerance compared to erect CMDs, as evidenced by a smaller increase in DEGs and DEMs with escalating drought levels and a more pronounced surge in flavonoid synthesis pathway metabolites. Collectively, these findings highlight the differences in drought responses between creeping and erect CMD varieties, offering insights into potential strategies for enhancing drought resilience in CMD plant species.

## Supporting information

S1 File List of qRT-PCR primers in this study. (DOCX)

S2 File PCA analysis of metabolites identified in D1 and D2. (DOCX)

S3 File Number of DEMs after D1 and D2 drought treatments. (DOCX)

S4 File Transcriptome quality control data, assembly data and functional annotation. (XLSX)

S1 Raw data (ZIP)
