# Supplementary material for: Integrative metabolomics and transcriptomics profiling reveals differential expression of flavonoid synthesis in Ophiopogon japonicus (L. f.) Ker-Gawl. in adaptation to drought
Source: PLoS One. 2025 Jan 7;20(1):e0313580. doi: 10.1371/journal.pone.0313580 (PMC11706389; doi:10.1371/journal.pone.0313580)
Supplement: S1 File — (DOCX) [file pone.0313580.s001.docx]

S1 List of qRT-PCR primers in this study

| Gene name | Primer sequence (5'-3') |
| --- | --- |
| actin-F | ATTCCCAAGGCAGCCACAA |
| actin-R | ATACAGACACCCAGCCTCCTTTA |
| DN4609301-F | CTCAATGCCTGCCAAACTGT |
| DN4609301-R | TGGTGCCTTACTTTATCCTCGGT |
| DN966021-F | CTACTTCCCTTAGATGTCCGTCG |
| DN966021-R | AAGGGTTCTTGTGGTCTGCTCT |
| DN2448301-F | GACTACTATTTCCTCCACCTTCTCC |
| DN2448301-R | CACAACTTTATTACCTCCCGTCC |
| DN1133803-F | CATCTCGTCAATAGCATTCTTCTCA |
| DN1133803-R | AGGAGCCTAACTTGGTGACCATAAT |
| DN10614501-F | GACAATCTGATAAAGAAGACGGGC |
| DN10614501-R | CTTGTACTTGTTGACGATCATGGC |
| DN878101-F | TTTTGAACTCCTCCTCCGTCCT |
| DN878101-R | ATACTGAAGAACTGCTACAATGCCT |
| DN3397101-F | GGGTCCCCAAGAAGAAAAAAT |
| DN3397101-R | CGATGGCGATCTTCTGGAA |
