# Supplementary material for: Integrative metabolomics and transcriptomics profiling reveals differential expression of flavonoid synthesis in Ophiopogon japonicus (L. f.) Ker-Gawl. in adaptation to drought
Source: PLoS One. 2025 Jan 7;20(1):e0313580. doi: 10.1371/journal.pone.0313580 (PMC11706389; doi:10.1371/journal.pone.0313580)
Supplement: S2 File — (DOCX) [file pone.0313580.s002.docx]

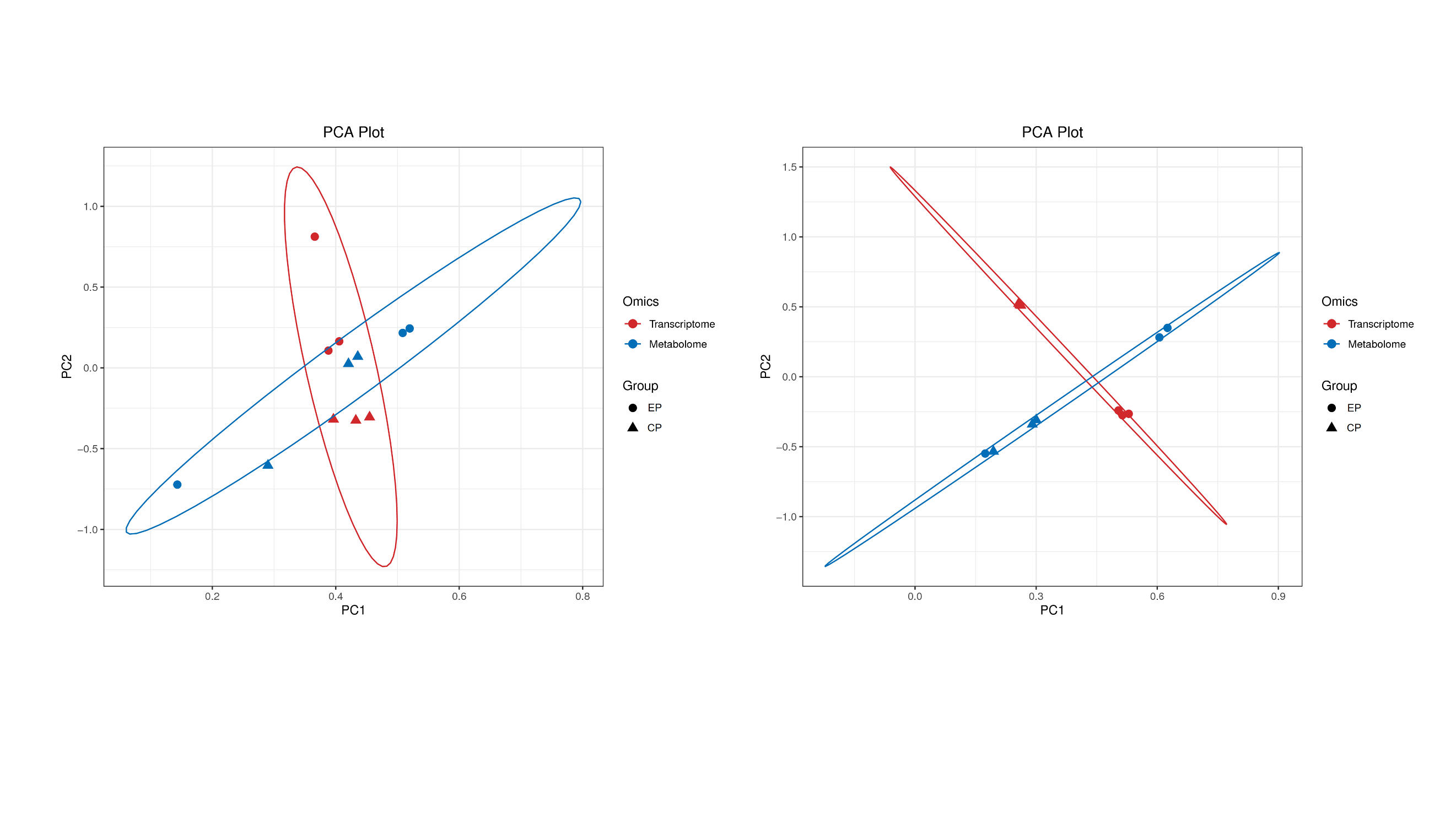
 (a) (b)

**S2** **PCA analysis of metabolites identified in D1 and D2.** Red color indicates transcriptome data, blue color indicates metabolome data, circle indicates EP, triangle indicates CP. (a) PCA analy-sis in the D1 treatment group, (b) PCA analysis in the D2 treatment group.
