# Supplementary material for: Integrative metabolomics and transcriptomics profiling reveals differential expression of flavonoid synthesis in Ophiopogon japonicus (L. f.) Ker-Gawl. in adaptation to drought
Source: PLoS One. 2025 Jan 7;20(1):e0313580. doi: 10.1371/journal.pone.0313580 (PMC11706389; doi:10.1371/journal.pone.0313580)
Supplement: S3 File — (DOCX) [file pone.0313580.s003.docx]

**S3.** **Number of differentially expressed metabolites after D1 and D2 drought treatments.** Red bars indicate an increase, blue bars indicate a decrease.
